# Calf-Level Factors Associated with Bovine Neonatal Pancytopenia – A Multi-Country Case-Control Study

**DOI:** 10.1371/journal.pone.0080619

**Published:** 2013-12-02

**Authors:** Bryony A. Jones, Carola Sauter-Louis, Joerg Henning, Alexander Stoll, Mirjam Nielen, Gerdien Van Schaik, Anja Smolenaars, Matthijs Schouten, Ingrid den Uijl, Christine Fourichon, Raphael Guatteo, Aurélien Madouasse, Simon Nusinovici, Piet Deprez, Sarne De Vliegher, Jozef Laureyns, Richard Booth, Jackie M. Cardwell, Dirk U. Pfeiffer

**Affiliations:** 1 Department of Population & Production Health, Royal Veterinary College, North Mymms, Herts, United Kingdom; 2 Department of Pathology and Infectious Diseases, Royal Veterinary College, North Mymms, Herts, United Kingdom; 3 Clinic for Ruminants, Faculty of Veterinary Science, University of Munich, Oberschleissheim, Germany; 4 School of Veterinary Science, University of Queensland, Gatton, Queensland, Australia; 5 Farm Animal Health, Faculty of Veterinary Medicine, Utrecht University, Utrecht, The Netherlands; 6 GD Animal Health Service, Deventer, The Netherlands; 7 UMR1300 Biology, Epidemiology and Risk Analysis in Animal Health, INRA, LUNAM Université, Oniris, Ecole nationale vétérinaire, agroalimentaire et de l’alimentation Nantes Atlantique Nantes, France; 8 Department of Internal Medicine and Clinical Biology of Large Animals, Faculty of Veterinary Medicine, Ghent University, Merelbeke, Belgium; 9 Department of Reproduction, Obstetrics and Herd Health, Faculty of Veterinary Medicine, Ghent University, Merelbeke, Belgium; Friedrich-Loeffler-Institut, Germany

## Abstract

Bovine neonatal pancytopenia (BNP), a high fatality condition causing haemorrhages in calves aged less than 4 weeks, was first reported in 2007 in Germany and subsequently observed at low incidence in other European countries and New Zealand. A multi-country matched case-control study was conducted in 2011 to identify calf-level risk factors for BNP. 405 BNP cases were recruited from 330 farms in Belgium, France, Germany and the Netherlands by laboratory confirmation of farmer-reported cases. Up to four calves of similar age from the same farm were selected as controls (1154 calves). Risk factor data were collected by questionnaire. Multivariable modelling using conditional logistic regression indicated that PregSure®BVD (PregSure, Pfizer Animal Health) vaccination of the dam was strongly associated with BNP cases (adjusted matched Odds Ratio - amOR 17.8 first lactation dams; 95% confidence interval – ci 2.4, 134.4; p = 0.005), and second or more lactation PregSure-vaccinated dams were more likely to have a case than first lactation vaccinated dams (amOR 2.2 second lactation; ci 1.1, 4.3; p = 0.024; amOR 5.3 third or more lactation; ci 2.9, 9.8; p = <0.001). Feeding colostrum from other cows was strongly associated with BNP if the dam was not PregSure-vaccinated (amOR 30.5; ci 2.1, 440.5; p = 0.012), but the effect was less if the dam was PregSure-vaccinated (amOR 2.1; ci 1.1, 4.0; p = 0.024). Feeding exclusively dam’s milk was a higher risk than other types of milk (amOR 3.4; ci 1.6, 7.5; p = 0.002). The population attributable fractions were 0.84 (ci 0.68, 0.92) for PregSure vaccination, 0.13 (ci 0.06, 0.19) for feeding other cows’ colostrum, and 0.15 (ci 0.08, 0.22) for feeding dam’s milk. No other calf-level factors were identified, suggesting that there are other important factors that are outside the scope of this study, such as genetics, which explain why BNP develops in some PregSure-colostrum-exposed calves but not in others.

## Introduction

Bovine neonatal pancytopenia (BNP) was first reported in 2007 in Germany and subsequently observed at low incidence in several other European countries and New Zealand [1]–[4]. It affects calves aged up to 4 weeks, causing skin and internal haemorrhages, prolonged haemorrhage from wounds or orifices, and high case fatality (90%) [5]–[7]. No evidence of an infectious, toxic or genetic aetiology has been found [2], [5]–[7]. In 2010 an association was suspected between affected calves and vaccination of their dams with PregSure®BVD (PregSure, Pfizer Animal Health) [8]. BNP was induced experimentally by feeding calves colostrum from unrelated PregSure-vaccinated dams that had previously given birth to BNP calves [9]. It was hypothesized that maternal antibodies in colostrum were destroying calf blood and bone marrow cells. PregSure is an inactivated bovine viral diarrhoea (BVD) virus type 1 vaccine, which was first marketed in 2004 [10]. The manufacturer, Pfizer Animal Health, voluntarily stopped sales to wholesalers in Europe in 2010, and New Zealand in 2011, pending investigations into the cause of BNP. The marketing authorisations in all concerned European Union (EU) Member States were suspended in August 2010 following an EU Commission decision based on recommendations from the European Medicines Agency Committee for Veterinary Medicinal Products (CVMP), with recall of product at wholesale level [11]. By the end of August 2012, 6913 suspected BNP cases had been reported by farmers, veterinarians and laboratories via the pharmacovigilance system in each European member state to the marketing authorization holder, Pfizer Animal Health. The numbers of reported suspected cases decreased in 2011 and 2012 compared with previous years. This case-control study was conducted to identify potential risk factors for BNP occurrence at the calf level.

## Materials and Methods

### Ethics Statement

All procedures on animals used in this study were in concordance with the ethical conditions for animal experimentation as mentioned in the European legislation (Directive 86/609/EEC). The blood samples collected from calves on the farms were taken for diagnostic purposes at the request of the owner as part of clinical veterinary practice and therefore were not considered to be experimental, so no formal approval of the protocol by an ethical committee in any of the four countries was required.

The study was conducted between January and December 2011 in four countries that had experienced a high number of BNP cases since 2007; Belgium, France, Germany and the Netherlands. The target was to recruit 400 cases and match them to 2–4 control calves of a similar age from the same farm. A multi-country design was required to obtain sufficient cases and to recruit cases from different management systems. A standard procedure for recruitment of cases and controls was developed jointly by the country research teams.

Affected farms were identified from suspected cases reported voluntarily by farmers and veterinarians to the research teams in the respective country. Case-reporting was encouraged via notices in the national veterinary and farming press in each country, asking for cases of calves aged less than one month old with one or more signs of bleeding from the skin (either spontaneously or from injection or ear tag sites), mucosal petechial haemorrhages, blood in diarrhoea, or death with internal or external bleeding. Free diagnostic testing and/or post mortem examination were offered.

All farms that reported suspected cases were visited by a veterinarian, either from the research team or their own veterinarian on behalf of the research team, who conducted a clinical examination. If the calf was less than 29 days of age and had one or more clinical signs of BNP; multiple skin haemorrhages, melena, petechiation of mucous membranes, or sudden death with internal haemorrhage, then it was included in the study as a suspected case. In order to confirm the diagnosis a whole blood sample was collected if the calf was alive, or if the calf was dead then a post mortem examination and bone marrow histopathology were performed. During the same visit, up to four calves without BNP clinical signs and aged 10–28 days were selected from the same farm to be matched controls. Whole blood samples were collected to verify that they did not have abnormal haematology. The veterinarian collected data on the characteristics of suspected case and the unaffected calves by face-to-face questionnaire-based interview (Questionnaire S1).

A case was therefore defined as a calf that had developed one or more BNP clinical signs on or before 28 days of age and had bone marrow depletion on histopathology and/or had thrombocytopenia (<150×10^9^/litre) and leucopenia (<5×10^9^/litre). A control was defined as a calf on the same farm as a case, aged 10–28 days at the time of case reporting, no clinical signs of BNP up to 28 days of age, and normal blood results (thrombocytes > = 300×10^9^/litre, leucocytes > = 5×10^9^/litre). Farmers were contacted again when control calves were 28 days of age to confirm that they had not developed BNP signs.

If the laboratory results subsequently indicated that a suspected case did not meet the case definition then the case and its matched controls were excluded from the study, and if a control calf did not have normal blood results then it was excluded from the study.

The questionnaire was developed in English, translated into French and German, and field-tested by researchers with experience of BNP cases from the four countries. In the Netherlands and Belgium the English questionnaire was used and the interview conducted in Dutch. There were 91 questions, of which 36 collected descriptive data on calf, dam and sire identification, calf characteristics, clinical signs and laboratory results, and 55 collected data on potential risk factors related to colostrum and milk feeding, dam and sire characteristics, and dam vaccination history. Data were entered into an internet-based form created in Open Source software (LimeSurvey http://www.limesurvey.org/), exported to Microsoft Excel and then to Stata IC 12.1 for coding, cleaning and analysis. Thirty (6 descriptive, 24 potential risk factor) questions were dropped due to a low number of responses or differences in interpretation between countries. Five variables identified the calf, dam and sire. Twenty two variables describing clinical and post-mortem signs and laboratory results were used to define cases and controls. Thirty four variables were used in the statistical analysis and an additional 15 variables were created by recoding, to give a total of 49 exposure variables (3 descriptive and 46 potential risk factor).

Due to the matched design, conditional logistic regression with farm as the matching variable was used for univariable analysis to obtain matched odds ratios (mOR), 95% confidence intervals (ci) and Wald test p values. It was first conducted on the dataset of 1559 calves, but due to missing observations the sample size for each variable was different. Variables with greater than 30% missing observations were excluded. The number of calves in the final multivariable model was 1296 due to missing observations in the retained variables, so univariable analysis was repeated with the smaller dataset. Variables with p values greater than 0.2 in univariable analysis were excluded from multivariable analysis. Pair-wise associations between the exposure variables were examined by the chi-squared test, and polychoric correlation was used to check for collinearity.

Multivariable analysis was conducted using conditional logistic regression with farm as the group variable. The model was built using forward stepwise regression starting with the variable with the highest odds ratio and lowest p value on univariable analysis. Variables were retained if the likelihood ratio test indicated that inclusion led to a better model fit (p<0.05). For variables from the same risk factor group (e.g. colostrum management, dam vaccination) that were likely to be collinear, the variable with the highest number of observations was added first. It was then removed and the other collinear variables were added and removed one by one. If more than one of the collinear variables improved the model then the one with the most observations and/or that was most biologically relevant was retained, as described below.

The population attributable fraction, PAF (proportion of cases in the total population that would be avoided if the exposure was removed, assuming the exposure is causal), was estimated using the *punafcc* package for Stata [12].

## Results

Data were collected for 502 suspected cases and 1583 potential controls from 410 farms. However, 62 suspected cases did not fit the case definition, 122 potential controls had incomplete blood results, and 210 potential controls had “atypical” blood results (thrombocytes <300×10^9^/litre and/or leucocytes <5×10^9^/litre). Exclusion of these led to the loss of 97 confirmed controls with no matching case and 35 confirmed cases with no matching control. The remaining 405 confirmed cases were matched to 1154 confirmed controls from 330 farms with an overall case control ratio of 1∶2.8 (Table 1). The proportions of dairy, beef and mixed farms that were included in the study from each country approximately reflected the proportions of those types of farms in each country. In the Netherlands, dairy farms predominate so almost all suspected calves came from dairy farms. In Germany a high proportion of cases recruited were from the south where beef farms are few so the high proportion of dairy farms reflects the regional distribution. The number of cases per farm varied from 1 to 3 and the number of controls from 1 to 9 (Table S1). The proportion of males amongst cases (41%) was higher than amongst controls (34%). In the Netherlands, cases were more likely than controls to be male probably due to the sale of male calves before one month of age so fewer were available to be selected as controls. This difference was not observed in the other countries. The most common breed of calf was Holstein-Friesian or Red Holstein-Friesian (56.0%), followed by Fleckvieh (13.7%), Belgian Blue (7.0%), Charolais (5.9%), other pure beef or dairy breeds (7.8%), and crossbreeds (9.6%) (Table S2). There was no evidence of a difference in breed distribution between cases and controls.

**Table 1:** Numbers of confirmed matched case and control calves per country.

	Belgium	France	Germany	Netherlands	Total
No. cases	82	103	87	133	405
No. controls	227	233	258	436	1154
Total	309	336	345	569	1559
Case-control ratio	1∶2.8	1∶2.3	1∶3.0	1∶3.3	1∶2.8
Total no. farms	63	92	75	100	330
- dairy	31	39	61	98	229
- beef	16	28	2	0	46
- mixed dairy and beef	16	25	12	2	55

### Univariable Analysis

#### Colostrum management (Table 2, Table S3)

There was no evidence of an association between case/control status and the following variables: *suckled dam within 12 hours of birth, time to first colostrum administration, number of times colostrum received in first 24 hours, artificial colostrum used*. There were increased odds of exposure of cases compared with controls for *total colostrum received, colostrum obtained from cow(s) different from dam, pooled colostrum, pooled colostrum includes colostrum of dam,* and *frozen colostrum*. These variables were associated with each other so *colostrum obtained from cow(s) different from dam* was included first in the multivariable model from this group because it had the highest number of observations, and pooled colostrum, *pooled colostrum includes colostrum of dam* and *frozen colostrum* were nested within it.

**Table 2:** Results of univariable analysis of colostrum and milk management variables showing variables with p<0.02 (n = 1296).

Variable (n)	Variable category	Matched odds ratio (mOR)	95% confidenceinterval	Wald test p value
*Total colostrum received by*	0–5	1.00		
*calf, litres (916)*	5.5–10	1.83	0.86, 3.88	0.12
	10.5–20	4.00	0.89, 18.00	0.07
	20.5–30	6.90	0.45, 106.20	0.17
	Ad lib	4.18	0.41, 42.95	0.23
*Colostrum obtained from*	No	1.00		
*cow(s) different from dam (1296)*	Yes	1.91	1.19, 3.05	0.007
*Pooled colostrum (from*	No	1.00		
*multiple cows) (1070)*	Yes	2.82	1.31, 6.09	0.008
*Pooled colostrum includes*	No	1.00		
*colostrum of dam (1061)*	Yes	2.20	0.95, 5.10	0.066
*Frozen colostrum (1133)*	No	1.00		
	Yes	1.75	0.83, 3.69	0.14
*Milk powder (1296)*	No	1.00		
	Yes	0.36	0.20, 0.66	0.001
*Raw milk from dam (1296)*	Other milk	1.00		
	Raw milk from dam	1.94	0.91, 4.14	0.085
*Type of milk fed to calf*	Milk powder	0.60	0.27. 1.31	0.20
*(1296)*	Raw milk mixed	1.00		
	Raw milk mixed & milk powder	0.34	0.11, 1.06	0.062
	Raw milk dam only	2.89	1.20, 6.97	0.018
	Raw milk dam only & milk powder	0.32	0.10, 1.06	0.062
*Raw milk from dam only*	No	1.00		
*(1296)*	Yes	4.13	1.78, 9.62	0.001

#### Milk feeding (Table 2, Table S3)

There were increased odds of exposure of cases compared with controls for feeding *milk powder, raw milk from dam, raw milk from dam only,* and *type of milk fed*. There was no evidence of an association between case/control status and feeding *raw milk*, *bulk milk, milk from cows with high somatic cell count/clinical mastitis* or *withdrawn/discarded milk. Type of milk fed* was included first in the multivariable model from this group because it provided the most information.

#### Dam and sire characteristics (Table 3, Table S4)

There was no evidence of an association between case/control status and *dam breed*, *dam was born on farm*, *dam was reared at another farm,* or *source of bull*. Case dams (dams of cases) had increased odds of being in second or more lactation rather than first lactation compared with control dams (dams of controls). Case dams had 12 times the odds of previously giving birth to a BNP calf compared to control dams (mOR 12.02; ci 5.44, 26.57; p = <0.001) Since *previously giving birth to a BNP calf* was considered to be on the causal pathway between other exposures and the outcome, it was not included in the multivariable model. *Lactation number* was correlated with many of the vaccination variables and was a potential confounder of the association between dam vaccination and case/control status. Cases had increased odds of having a Fleckvieh rather than a Holstein-Friesian or Red Holstein-Friesian sire, compared with controls. It was not possible to obtain estimates for all breeds because of low numbers of observations.

**Table 3:** Results of univariable analysis of dam and sire characteristics showing variables with p<0.02 (n = 1296).

Variable (n)	Variable category	Matched odds ratio (mOR)	95% confidence interval	Wald test p value
*Lactation number*	1	1.00		
*(1296)*	2	2.30	1.41, 3.74	0.001
	3+	6.16	4.00, 9.49	<0.001
*Dam had previous*	No	1.00		
*BNP calf (1245)*	Yes	12.02	5.44, 26.57	<0.001
*Bull Breed (1223)*	Belgian Blue	0.86	0.43, 1.71	0.66
	Holst Friesian/Red HF	1.00		
	Fleckvieh	4.31	1.02, 18.10	0.046
	Other pure breeds*	1.50	0.57, 3.96	0.42
	Crossbreeds	2.70	0.47, 15.62	0.27

* Brown Swiss, Limousin, Limpurger, Pinzgau, Charolais, MRIJ, Montbéliarde, Abondance, Scandinavian Roodbont, Aubrac, Angus, Blanc Bleu, Maine Anjou, Normande, Prog Federat Eur Pie, Blonde d’Aquitaine, Aure et St Girons Ca.

#### Dam BVD vaccination (Table S5)

Case dams had increased odds of being vaccinated against BVD compared with control dams, and increased odds of having received more doses of BVD vaccine. There was no evidence of any difference in the timing of last BVD vaccination before calving between case and control dams. BVD vaccination variables (all types of vaccine) were correlated with each other, as well as with specific BVD vaccine variables. They were not used in the multivariable model because the specific BVD vaccine variables provided more information.

#### Dam PregSure vaccination (Table 4, Table S5)

Case dams had increased odds of being PregSure-vaccinated compared with control dams. Case dams were more likely to have received their last dose of PregSure longer before calving, and to have received more doses of PregSure, compared with control dams. PregSure vaccination variables were correlated with each other so they were added to the model separately. The responses to *dam PregSure-vaccinated* were more reliable than *no. doses PregSure* because some farmers reported the initial two doses as a single dose, and for some dams the number of doses was unknown. First lactation cows were less likely to have received PregSure (49%) compared with second (14%) and third or more lactation cows (12%), and were more likely to have received fewer doses of PregSure than second or third or more lactation cows (Table S6). The polychoric correlation coefficient between PregSure doses and lactation number was 0.61.

**Table 4:** Results of univariable analysis of dam vaccination variables showing variables with p<0.02 (n = 1296).

Variable (n)	Variable category	Matched oddsratio (mOR)	95% confidenceinterval	Wald testp value
*Dam PregSure*	No	1.00		
*vaccinated (1296)*	Yes	16.23	6.95, 37.93	<0.001
*No. doses PregSure*	0	1.00		
*(1296)*	1–2	7.17	2.93, 17.53	<0.001
	3–4	31.02	12.54, 76.73	<0.001
	5–8	42.11	15.23, 116.42	<0.001
	unknown doses	1.35	0.30, 6.13	0.70
*No. mths before*	>1–3	1.43 per	0.89, 2.28	0.14
*calving PregSure*	>3–6	change in		
*vaccinated (935)*	>6–12	level		
	>12–24			
	>24–36			
	>36–75			
*Bovilis BVD (1287)*	No	1.00		
	Yes	2.14	0.73, 6.23	0.17
*Bovidec BVD (1284)*	No	1.00		
	Yes	4.00	0.93, 17.20	0.063
*Mucosiffa BVD (1290)*	No	1.00		
	Yes	2.91	0.67, 12.71	0.15
*Bluetongue*	No	1.00		
*vaccination (1256)*	Yes	2.80	1.25, 6.27	0.013
*IBR vaccination*	No	1.00		
*(1237)*	Yes	2.76	0.78, 9.81	0.12
*No. diseases*	0	1.41 per	1.02, 1.94	0.037
*vaccinated against*	1	change in		
*apart from BVD*	2	level		
*(1171)*	3			
	4			
	5			

#### Other dam BVD vaccinations (Table 4, Table S5)

After PregSure, Bovilis BVD (MSD Animal Health) was the most commonly used BVD vaccine, followed by Rispoval 3 (containing BVD, Respiratory Syncytial Virus – RS, and Parainfluenza type 3, Pfizer Animal Health) and Bovidec BVD (Novartis Animal Health). Case dams were more likely to have received Bovilis BVD, Bovidec BVD and Mucosiffa (Merial) than control dams. There was no evidence of an association between case/control status and Rispoval BVD, Rispoval RS-BVD, Rispoval 3 or Mucobovin (Merial) vaccination of dams. Some of these variables were correlated with BVD vaccination (all types of vaccine) and PregSure variables. There were 26 BVD vaccine combinations and 34 BVD vaccination sequences (Tables S7, S8). The most common combination was PregSure and Bovilis BVD (22%) and the most common sequence of BVD vaccines was PregSure followed by Bovilis BVD (21%). When the BVD vaccine combinations were regrouped into five categories (Table S5), case dams were more likely to have received PregSure in combination with other BVD vaccines than PregSure only, compared with control dams. When the BVD vaccine sequence variable was regrouped into eight categories (Table S5), case dams had increased odds of having received one or more other BVD vaccine then PregSure, or receiving one or more other BVD vaccine, then PregSure, then one or more other BVD vaccine, rather than PregSure only, compared with control dams. These variables were correlated with the other BVD vaccination variables so they were added separately to the model.

#### Other dam vaccinations (Table 4, Table S5)

Case dams had increased odds of having received bluetongue or IBR vaccination, and being vaccinated against more diseases, compared with control dams. There was no association between case/control status and dams receiving rota/coronavirus or other vaccines. These variables were correlated with each other and with some of the other vaccine variables.

### Multivariable Analysis

Four variables were retained in the final model: *dam PregSure-vaccinated, colostrum from different cow(s), lactation number,* and *raw milk from dam only* (Table 5, Table S9). *No. doses PregSure,* and combinations and sequences of BVD vaccines were alternatives to *dam PregSure-vaccinated, and frozen colostrum*, *pooled colostrum* and *pooled colostrum including dam’s* were alternatives to c*olostrum from different cow(s),* but they had fewer observations.

**Table 5:** Results of multivariable model (n = 1296); conditional logistic regression model with farm as matching variable.

Exposure variable		Adjusted matched odds ratio (amOR)	95% confidence interval	Wald test P value
Dam PregSure vaccination,	Unvaccinated	1.0		
no colostrum from different cow(s), dam is first lactation	Vaccinated	17.8	2.4, 134.4	0.005
Dam PregSure vaccination,	Unvaccinated	1.0		
calf received colostrum from different cow(s), dam is first lactation	Vaccinated	1.2	0.3, 5.6	0.79
Dam PregSure vaccination,	Unvaccinated	1.0		
no colostrum from different cow(s), dam is second lactation	Vaccinated	86.0	7.4, 995.3	<0.001
Dam PregSure vaccination,	Unvaccinated	1.0		
no colostrum from different cow(s), dam is third or more lactation	Vaccinated	132.0	9.9, 1764.7	<0.001
Dam lactation no.	1	1.0		
when dam is unvaccinated	2	0.5	0.1, 1.6	0.23
	3+	0.7	0.2, 2.4	0.60
Dam lactation no.	1	1.0		
when dam is PregSure vaccinated	2	2.2	1.1, 4.3	0.024
	3+	5.3	2.9, 9.8	<0.001
Colostrum from different cow(s)	No	1.0		
when dam is unvaccinated	Yes	30.5	2.1, 440.5	0.012
Colostrum from different cow(s)	No	1.0		
when dam is PregSure vaccinated	Yes	2.1	1.1, 4.0	0.024
Raw milk from dam only	No	1.0		
	Yes	3.4	1.6 7.5	0.002

Exposure variables are dam PregSure vaccination, lactation number, colostrum from different cow(s) and raw milk from dam only. Interactions were included between dam PregSure vaccination and lactation number, and between dam PregSure vaccination and colostrum from different cow(s). The interaction terms have been multiplied with the baseline odds ratios to obtain odds ratios for each variable category above baseline.

There was evidence of interaction between *dam PregSure-vaccinated* and *lactation number,* between *dam PregSure-vaccinated* and *colostrum from different cow(s)*, and between *lactation number* and *colostrum from different cow(s)*. Including two-way interactions between *dam PregSure-vaccinated* and *lactation number* and between *dam PregSure-vaccinated* and *colostrum from different cow(s)* was a better fit than the models with single interactions or all three interactions. The odds of a case having a second lactation dam were five times the odds of having a first lactation dam, if the dam was PregSure-vaccinated (interaction term adjusted matched odds ratio - amOR 4.8; ci 1.1, 20.7; p = 0.034), and the odds of having a vaccinated third or more lactation dam were 7 times the odds of having a vaccinated first lactation dam (interaction term amOR 7.4; ci 1.9, 28.9; p = 0.004). The odds of a case having received colostrum from different cow(s) were 90% lower than the odds of not having received colostrum from other cows, if the dam was PregSure-vaccinated (interaction terms amOR 0.1; ci 0.01, 0.95; p = 0.046).

There was very strong evidence of an association between case/control status and having a PregSure-vaccinated dam. A case had 18 times the odds of being born to a dam that was PregSure-vaccinated rather than unvaccinated compared with a control, if the dam was first lactation and the calf did not receive colostrum from other cows, adjusting for type of milk fed (amOR 17.8; ci. 2.4, 134.4; p = 0.005). For calves that received colostrum from other cows, there was no evidence of a difference in the odds of a case having a PregSure-vaccinated dam rather than an unvaccinated dam (amOR 1.2; ci 0.3, 5.6; p = 0.79), if the dam was first lactation, adjusting for type of milk fed. Compared with a control, a case with a second lactation dam that did not receive colostrum from other cows had 86 times the odds of having a PregSure-vaccinated dam rather than an unvaccinated dam (amOR 86.0; ci 7.4, 995.3; p = <0.001), and a case with a third or more lactation dam that did not receive colostrum from other cows had 132 times the odds of having a PregSure-vaccinated dam rather than an unvaccinated dam (amOR 132.0; ci 9.9, 1764.7; p = <0.001), adjusting for type of milk fed.

For calves with unvaccinated dams, there was no evidence of a difference in the odds of a case being born to a dam in first, second or third or more lactation compared with a control, adjusting for source of colostrum and type of milk fed (second lactation amOR 0.5; ci 0.1, 1.6; p = 0.23, third or more lactation amOR 0.7; ci 0.2, 2.4; p = 0.60). However, if the dam was PregSure-vaccinated, a case had twice the odds of having a second lactation dam (amOR 2.2; ci 1.1, 4.3; p = 0.024) and 5 times the odds of having a third lactation dam (amOR 5.3; ci 2.9, 9.8; p = <0.001) compared with a first lactation dam, adjusting for source of colostrum and type of milk fed.

A case had 30 times the odds of having received colostrum from another dam compared with a control, if its dam was not PregSure-vaccinated (amOR 30.5; ci 2.1, 440.5; p = 0.012), but had only twice the odds of having received colostrum from another dam if its dam had been PregSure-vaccinated, adjusting for lactation number and type of milk fed (amOR 2.1; ci 1.1, 4.0; p = 0.024).

A case had 3 times the odds of having been fed raw milk only from its dam rather than other types of milk (with or without dams milk) compared with a control (amOR 3.4; ci 1.6, 7.5; p = 0.002) when adjusting for dam PregSure-vaccination, lactation number and source of colostrum.

The population attributable fraction (PAF) for *dam PregSure vaccination* was 0.84 (ci 0.68, 0.92) indicating that if no PregSure-vaccinated cows had been used for breeding then 84% of cases would have been avoided. If calves had been fed colostrum from their own dams only, rather than colostrum from other cows, then 12% of cases would have been avoided (PAF 0.13; ci 0.06, 0.19). If calves had not been fed exclusively on their dam’s milk, then 15% of cases would have been avoided (PAF 0.15; ci 0.08, 0.22). These estimates are based on the assumption of a causal relationship between each variable and case/control status.

Of the 440 confirmed BNP cases in this study, 20 cases (4.5%) had dams that were not PregSure-vaccinated and had not previously had a BNP calf, had received only dam’s colostrum, and came from farms with no history of PregSure vaccination or BNP cases.

## Discussion

Our results show that PregSure vaccination of a cow was strongly associated with her having a BNP calf, and that older PregSure-vaccinated cows were more likely to have a BNP calf than younger vaccinated cows. This was partly explained by the increased odds of BNP with increasing doses of PregSure and correlation between PregSure doses and lactation number. Feeding colostrum from other cows, in addition to or instead of dam colostrum, was strongly associated with BNP if the dam had not been PregSure-vaccinated, but its effect was less if the dam had been vaccinated. When calves received colostrum from other cows, most farmers were unable to identify which cows the colostrum came from, so their PregSure vaccination status was unknown. These findings suggest that if a dam had been PregSure-vaccinated and the calf received colostrum from its dam then there was an increased odds of BNP. But if the calf received some colostrum from other cows (which may or may not have been vaccinated) then the dilution effect of receiving some “non-BNP colostrum” reduced the odds. However, for calves of unvaccinated cows, feeding colostrum from other cows was strongly associated with BNP, presumably because feeding colostrum from multiple cows increased the chance of the calf ingesting some colostrum from a PregSure-vaccinated cow. The strong association between BNP and PregSure vaccination, and consumption of colostrum from PregSure-vaccinated dams, was consistent with single-country case-control studies with small sample sizes conducted in the UK and Germany [13], [14]. We also found that exclusively feeding dam’s raw milk was associated with an increased odds of BNP, compared to feeding milk from one or more other sources. Early lactation milk contains some antibodies that can still be absorbed up to 48 hours after birth [15]. Feeding milk powder or bulk tank milk instead of, or to supplement, dam’s milk will reduce the amount of antibody ingested. Alternatively the observed effect could be due to an association between feeding dam’s milk and other management factors that affect the risk of BNP. In our study, calves that suckled their dam within 12 hours of birth were more likely to be fed only dam’s raw milk. We hypothesize that calves that suckle could receive a larger volume of colostrum earlier in life leading to higher levels of antibody absorption, which could increase the risk of BNP.

The estimated population attributable fractions indicate that the most effective intervention would have been to avoid breeding from PregSure-vaccinated cows (86% case avoided). Not exclusively feeding dam’s milk would have avoided 15% cases and not feeding colostrum from other cows would have avoided 12%.

Twenty cases in this study had no apparent exposure to the identified risk factors. They could have been misclassified with respect to dam vaccination status, colostrum feeding or dam’s BNP history due to incorrect farmer recall, or they could have developed pancytopenia due to other causes. Prior to the identification of BNP there were sporadic cases of unexplained pancytopenia in young calves [16]–[19], so some of our 20 calves could represent a background incidence of pancytopenia that is unrelated to ingestion of colostrum from PregSure-vaccinated cows. Further research is required to determine whether sporadic unexplained pancytopenia cases have the same pathogenesis as PregSure-associated BNP cases, and therefore whether the introduction of PregSure vaccination has increased the incidence of an existing but rare syndrome.

Despite widespread use of PregSure vaccine prior to its withdrawal, BNP incidence has been low, suggesting that consumption of colostrum from a PregSure-vaccinated dam is not a sufficient cause of BNP. We did not identify any other important calf or management-related risk factors. Research into BNP pathogenesis is on-going with a prevailing hypothesis that it is a neonate-maternal incompatibility phenomenon related to PregSure-induced maternal alloantibodies against bovine cell surface molecules due to bioprocess-related impurities from the cell line used for virus propagation [20]–[22]. Bastian et al. [20] showed that sera of dams that had previously had a BNP calf contained alloantibodies that bound to bovine leucocytes, and Foucras et al [23] reproduced BNP in healthy calves by transferring serum antibodies from PregSure-vaccinated dams. Animals vaccinated with three doses of PregSure had higher alloantibody titres compared with animals receiving a single PregSure dose or other BVD vaccines [10]. This supports our finding that the odds of BNP increase with the number of PregSure doses given to the dam. Bridger et al [24] showed that maternal alloantibodies to surface antigens of neonatal leucocytes are transferred via colostrum from BNP dams to neonatal calves, and higher antibody titres in the dam led to more severe clinical signs in the calf. Various proteins found in both PregSure vaccine and the cell line used to produce the vaccine have been implicated as possible alloantigen candidates [21]–[23], [25]. Bell et al. [26] suggest that feeding BNP colostrum from multiple cows increases the likelihood that the colostrum will contain antibodies that will react with most calf allotypes, which fits with our finding that calves from unvaccinated dams were at increased risk of BNP if they were fed colostrum from other cows. They suggest that the unique adjuvant in PregSure could amplify production of alloantibody against vaccine antigens as well as boosting pregnancy-induced maternal alloantibodies against paternally-derived foetal MHC antigens [26]. The latter mechanism may explain the aetiology of some of the sporadic cases of unexplained pancytopenia that are not linked to the ingestion of colostrum from PregSure-vaccinated cows.

It was anticipated that BNP incidence would decline during the study period due to withdrawal of PregSure from distribution in 2010, and advice given to some farmers to avoid feeding colostrum from cows that had previously had BNP calves. A multi-country study was therefore necessary to obtain sufficient BNP cases to have the statistical power to detect important risk factors. A case-control study is the most appropriate design to investigate a rare disease, and for the investigation of calf-level risk factors it was necessary to match by farm. This meant that most management factors were the same for both cases and controls on the same farm, and, even where there were differences, the farmer could have reported routine practices rather than what had happened to individual calves, leading to misclassification and an underestimation of effect. The multi-country design meant that there was variation between countries in case-reporting, farm visits, sample collection, post-mortem examination, laboratory testing and questionnaire interpretation. There were also differences between countries in production methods, cattle breeds, and policies on BVD control and vaccination programmes. The matched design minimised the effect of these country differences on our results.

Case recruitment relied on passive reporting by farmers and veterinarians, strengthened by a communication campaign to encourage reporting of cases and provision of free post mortem examination and laboratory testing. The reasons for a farmer or veterinarian not reporting a case might include; being unaware of the invitation to report cases or too busy to report, or having reported cases prior to the study and therefore seeing no benefit in reporting further cases. It is possible that farmers who report disease are more likely to take preventive measures such as vaccination. There is therefore a potential bias in selection of case farms in that the study population might not be representative of all BNP-affected farms in the four countries. But even if these biases were present, they are unlikely to have biased the particular inferences reported here.

Strict definitions for case and control calves were applied due to variations between countries in time elapsed before blood analysis and use of multiple laboratories, to minimise the inclusion of false positive cases. Using the thrombocyte and leucocyte values of the Netherlands control calves, a 95% reference interval was calculated based on the mean and standard deviation, where values below the lower limit of the interval were considered abnormal for the current study. Using these results the blood values for controls were set at >300×10^9^/litre thrombocytes and >5×10^9^/litre leucocytes. For case calves, the thrombocyte value was set at <150×10^9^/litre, the lower threshold of the reference range in the Netherlands.

The number of questions in the questionnaire was high, to capture management practices in the diverse farming systems of the four countries, which increased the risk of detecting an association by chance when there was no true association. As the questionnaire had been translated into three languages and administered by a range of veterinarians and researchers, prior to data analysis the research team discussed in detail how the questionnaire had been administered and differences in interpretation, and there was regular consultation during analysis to inform the interpretation of results.

In conclusion, this multi-country study provides strong evidence that receiving colostrum from a PregSure-vaccinated cow is a major risk factor for BNP. If calves are only given colostrum from unvaccinated cows then it is highly unlikely that a calf will develop BNP. The study design and sample size provided adequate statistical power to investigate many hypotheses related to farming practices such as colostrum management, calf feeding and vaccination, but no other important calf management-related risk factors were identified. This suggests that there are other important factors, such as genetics, that were outside the scope of this study, which explain why BNP develops in some PregSure-colostrum-exposed calves but not in others. These require further investigation.

## Supporting Information

Table S1 Number of cases and controls per farm: number (percentage) of farms with each case:control ratio.
(DOCX)

Table S2 Numbers of cases and controls by breed.
(DOCX)

Table S3 Results of descriptive and univariable analysis of colostrum and milk management variables.
(DOCX)

Table S4 Results of descriptive and univariable analysis of dam and sire characteristics.
(DOCX)

Table S5 Results of descriptive and univariable analysis of dam vaccination variables.
(DOCX)

Table S6 Number of PregSure doses by lactation number of cow.
(DOCX)

Table S7 Combinations of BVD vaccines.
(DOCX)

Table S8 Sequences of BVD vaccines.
(DOCX)

Table S9 Multivariable model Stata output.
(DOCX)

Questionnaire S1 (DOCX)
