# Supplementary material for: Calf-Level Factors Associated with Bovine Neonatal Pancytopenia – A Multi-Country Case-Control Study
Source: PLoS One. 2013 Dec 2;8(12):e80619. doi: 10.1371/journal.pone.0080619 (PMC3846664; doi:10.1371/journal.pone.0080619)
Supplement: Table S1 — Number of cases and controls per farm: number (percentage) of farms with each case:control ratio. (DOCX) [file pone.0080619.s001.docx]

*Table S1. Number of cases and controls per farm: number (percentage) of farms with each case:control ratio*

|  |  | Number of controls per farm | | | | | | | | |  |
| --- | --- | --- | --- | --- | --- | --- | --- | --- | --- | --- | --- |
|  |  | 1 | 2 | 3 | 4 | 5 | 6 | 7 | 8 | 9 | Total |
| Number of cases per farm | 1 | 26 (8) | 46 (14) | 92 (28) | 89 (27) | 1 (0.3) | 2 (0.6) | 1 (0.3) | 1 (0.3) | 0 | 258 (78) |
|  | 2 | 4 (1) | 5 (2) | 10 (3) | 9 (3) | 5 (2) | 11 (3) | 13 (4) | 11 (3%) | 1 (0.3) | 69 (21) |
|  | 3 | 0 | 0 | 1 (0.3) | 0 | 2 (0.6) | 0 | 0 | 0 | 0 | 3 (1) |
|  | Total | 30 (9) | 51 (15) | 103 (31) | 98 (30) | 8 (2) | 13 (4) | 14 (4) | 12 (4) | 1 (0.3) | 330 (100) |
