# Supplementary material for: Calf-Level Factors Associated with Bovine Neonatal Pancytopenia – A Multi-Country Case-Control Study
Source: PLoS One. 2013 Dec 2;8(12):e80619. doi: 10.1371/journal.pone.0080619 (PMC3846664; doi:10.1371/journal.pone.0080619)
Supplement: Table S2 — Numbers of cases and controls by breed. (DOCX) [file pone.0080619.s002.docx]

*Table S2 Numbers of cases and controls by breed*

|  | Belgium | | France | | Germany | | Netherlands | | All | | |
| --- | --- | --- | --- | --- | --- | --- | --- | --- | --- | --- | --- |
| Breed | Cases | Controls | Cases | Controls | Cases | Controls | Cases | Controls | Cases | Controls | Total |
| Belgian Blue | 28  (34.1%) | 81 (35.8%) | 0 | 0 | 0 | 0 | 0 | 0 | 28  (7.0%) | 81  (7.1%) | 109 (7.0%) |
| Holstein Friesian/Red Holstein Friesian | 45  (54.9%) | 126 (55.8%) | 41 (41.4%) | 86 (37.9%) | 19 (21.8%) | 60 (23.3%) | 112 (84.2%) | 378 (86.7%) | 217 (54.1%) | 650 (56.7%) | 867 (56.0%) |
| Fleckvieh | 0 | 0 | 0 | 0 | 53 (60.9%) | 159 (61.6%) | 0 | 0 | 53 (13.2%) | 159 (13.9%) | 212 (13.7%) |
| Charolais | 0 | 0 | 27 (27.2%) | 64 (28.2%) | 0 | 0 | 0 | 0 | 27  (6.7%) | 64  (5.6%) | 91  (5.9%) |
| Other pure breeds* | 0 | 0 | 23 (23.2%) | 57 (25.1%) | 9  (10.3%) | 24  (9.3%) | 2  (1.5%) | 6  (1.4%) | 34  (8.5%) | 87  (7.6%) | 121 (7.8%) |
| Crossbreeds | 9  (11.0%) | 19  (8.4%) | 8  (8.1%) | 20  (8.8%) | 6  (6.9%) | 15  (5.8%) | 19 (14.3%) | 52 (11.9%) | 42  (10.5%) | 106  (9.2%) | 148 (9.6%) |
| Total | 82 | 226 | 99 | 227 | 87 | 258 | 133 | 436 | 401 | 1147 | 1548 |

* Brown Swiss, Limousin, Montbéliarde, Normande, Limpurger, Pinzgau, Meuse Rhine Ijssel (MRIJ), Abondance, Aubrac, Brune, Maine Anjou
