# Supplementary material for: Calf-Level Factors Associated with Bovine Neonatal Pancytopenia – A Multi-Country Case-Control Study
Source: PLoS One. 2013 Dec 2;8(12):e80619. doi: 10.1371/journal.pone.0080619 (PMC3846664; doi:10.1371/journal.pone.0080619)
Supplement: Table S3 — Results of descriptive and univariable analysis of colostrum and milk management variables. (DOCX) [file pone.0080619.s003.docx]

*Table S3 Results of descriptive and univariable analysis of colostrum and milk management variables (n= 1296)*

| **Variable** | **n** | **% missing** | **Variable category** | **No. cases (%)** | **No. controls**  **(%)** | **Matched odds ratio (mOR)** | **95% confidence interval** | **Wald test p value** |
| --- | --- | --- | --- | --- | --- | --- | --- | --- |
| *Suckled dam within 12 hours of birth* | 1247 | 4 | Yes | 76 (23%) | 182 (20%) | 1.00 |  |  |
|  |  |  | Possibly | 51 (15%) | 150 (16%) | 0.90 | 0.44, 1.84 | 0.78 |
|  |  |  | No | 203 (62%) | 585 (64%) | 0.82 | 0.42, 1.62 | 0.58 |
| *Time to first colostrum administration, if separated from dam at birth (hours)* | 1022 | 21 | 0-3 | 197 (72%) | 562 (75%) | 1.00 |  |  |
|  |  |  | 3.5-6 | 58 (21%) | 140 (19%) | 1.01 | 0.59, 1.75 | 0.97 |
|  |  |  | 6.5-12 | 14 (5%) | 44 (6%) | 0.63 | 0.24, 1.65 | 0.35 |
|  |  |  | 12.5-48 | 3 (1%) | 4 (1%) | - | - | - |
| *No. times colostrum received in first 24 hours* | 924 | 29 | 1-3 | 222 | 627 | 1.00 | 0.25, 3.64 | 0.95 |
|  |  |  | 4-8 | 18 (8%) | 57 (8%) | 0.96 |  |  |
| *Total colostrum received by calf*, litres | 916 | 29 | 0-5 | 102 (43%) | 275 (41%) | 1.00 |  |  |
|  |  |  | 5.5-10 | 90 (38%) | 266 (39%) | 1.83 | 0.86, 3.88 | 0.12 |
|  |  |  | 10.5-20 | 37 (15%) | 113 (17%) | 4.00 | 0.89, 18.00 | 0.07 |
|  |  |  | 20.5-30 | 2 (1%) | 4 (1%) | 6.90 | 0.45, 106.20 | 0.17 |
|  |  |  | Ad lib | 8 (3%) | 19 (3%) | 4.18 | 0.41, 42.95 | 0.23 |
| *Colostrum obtained from cow(s) different from dam* | 1296 | 0 | No | 271 | 820 | 1.00 | 1.19, 3.05 | 0.007 |
|  |  |  | Yes | 69 (20%) | 136 (14%) | 1.91 |  |  |
| *Pooled colostrum (from multiple cows)* | 1070 | 17 | No | 247 | 714 | 1.00 | 1.31, 6.09 | 0.008 |
|  |  |  | Yes | 38 (13%) | 71 (9%) | 2.82 |  |  |
| *Pooled colostrum includes colostrum of dam* | 1061 | 18 | No | 246 | 706 | 1.00 | 0.95, 5.10 | 0.066 |
|  |  |  | Yes | 38 (13%) | 71 (9%) | 2.20 |  |  |
| *Frozen colostrum* | 1133 | 13 | No | 268 | 777 | 1.00 | 0.83, 3.69 | 0.14 |
|  |  |  | Yes | 33 (11%) | 55 (7%) | 1.75 |  |  |
| *Artificial colostrum (replacer) used* | 1059 | 18 | No | 275 | 768 | 1.00 | 0.14, 4.83 | 0.83 |
|  |  |  | Yes | 5 (2%) | 11 (1%) | 0.82 |  |  |
| *Milk powder* | 1296 | 0 | No | 211 | 514 | 1.00 |  |  |
|  |  |  | Yes | 129 (38%) | 442 (46%) | 0.36 | 0.20, 0.66 | 0.001 |
| *Raw Milk* | 1296 | 0 | No | 89 | 292 | 1.00 |  |  |
|  |  |  | Yes | 251 (74%) | 664 (69%) | 1.45 | 0.68, 3.05 | 0.33 |
| *Raw milk from dam* | 1296 | 0 | Other milk | 164 | 512 | 1.00 |  |  |
|  |  |  | Raw milk from dam | 176 (52%) | 444 (46%) | 1.94 | 0.91, 4.14 | 0.085 |
| *Bulk milk* | 1296 | 0 | Other milk | 241 | 689 | 1.00 |  |  |
|  |  |  | Bulk milk | 99 (29%) | 267 (28%) | 1.17 | 0.55, 2.53 | 0.68 |
| *Milk from cows with high SCC or clinical mastitis* | 1296 | 0 | Other milk | 276 | 777 | 1.00 |  |  |
|  |  |  | High SCC/mastitis milk | 64 (19%) | 179 (19%) | 1.09 | 0.44, 2.68 | 0.86 |
| *Withdrawn/discarded milk* | 1296 | 0 | Other milk | 290 | 802 | 1.00 |  |  |
|  |  |  | Withdrawn/discarded milk | 50 (15%) | 154 (16%) | 0.58 | 0.25, 1.36 | 0.21 |
| *Type of milk fed to calf* | 1296 | 0 | Milk powder | 89 (26%) | 292 (31%) | 0.60 | 0.27. 1.31 | 0.20 |
|  |  |  | Raw milk mixed | 138 (41%) | 370 (39%) | 1.00 |  |  |
|  |  |  | Raw milk mixed & milk powder | 21 (6%) | 76 (8%) | 0.34 | 0.11, 1.06 | 0.062 |
|  |  |  | Raw milk dam only | 73 (21%) | 144 (15%) | 2.89 | 1.20, 6.97 | 0.018 |
|  |  |  | Raw milk dam only & milk powder | 19 (6%) | 74 (8%) | 0.32 | 0.10, 1.06 | 0.062 |
| *Raw milk from dam only* | 1296 | 0 | No | 267 | 812 | 1.00 |  |  |
|  |  |  | Yes | 73 (21%) | 144 (15%) | 4.13 | 1.78, 9.62 | 0.001 |
