# Supplementary material for: Calf-Level Factors Associated with Bovine Neonatal Pancytopenia – A Multi-Country Case-Control Study
Source: PLoS One. 2013 Dec 2;8(12):e80619. doi: 10.1371/journal.pone.0080619 (PMC3846664; doi:10.1371/journal.pone.0080619)
Supplement: Table S4 — Results of descriptive and univariable analysis of dam and sire characteristics. (DOCX) [file pone.0080619.s004.docx]

*Table S4 Results of descriptive and univariable analysis of dam and sire characteristics (n=1296)*

| **Variable** | **n** | **% missing** | **Variable category** | **No. cases (%)** | **No. controls**  **(%)** | **Matched odds ratio (mOR)** | **95% confidence interval** | **Wald test p value** |
| --- | --- | --- | --- | --- | --- | --- | --- | --- |
| *Dam Breed* | 1290 | 0.5 | Belgian Blue | 29 (9%) | 82 (9%) | 0.57 | 0.20, 1.64 | 0.30 |
|  |  |  | HF/Red HF | 205 (61%) | 586 (62%) | 1.00 |  |  |
|  |  |  | Fleckvieh | 39 (12%) | 112 (12%) | 1.68 | 0.23, 12.46 | 0.61 |
|  |  |  | Other pure breeds* | 43 (13%) | 108 (11%) | 0.63 | 0.19, 2.12 | 0.45 |
|  |  |  | Crossbreeds | 22 (7%) | 64 (7%) | 1.06 | 0.55, 2.04 | 0.87 |
| *Lactation number* | 1296 | 0 | 1 | 31 (9%) | 284 (30%) | 1.00 |  |  |
|  |  |  | 2 | 61 (18%) | 238 (25%) | 2.30 | 1.41, 3.74 | 0.001 |
|  |  |  | 3+ | 248 (73%) | 434 (45%) | 6.16 | 4.00, 9.49 | <0.001 |
| *Calf by embryo transfer* | 1245 | 4 | No | 337 | 897 | 1.00 |  |  |
|  |  |  | Yes | 3 (1%) | 8 (1%) | 1.12 | 0.22, 5.79 | 0.89 |
| *Dam born on farm* | 1282 | 1 | No | 19 | 57 | 1.00 |  |  |
|  |  |  | Yes | 318 (94%) | 888 (94%) | 1.08 | 0.56, 2.07 | 0.82 |
| *Dam reared at other farm* | 1296 | 0 | No | 316 | 888 | 1.00 |  |  |
|  |  |  | Yes | 24 (7%) | 68 (7%) | 1.55 | 0.55, 4.33 | 0.41 |
| *Dam had previous BNP calf* | 1245 | 4 | No | 308 | 897 | 1.00 |  |  |
|  |  |  | Yes | 32 (9%) | 8 (1%) | 12.02 | 5.44, 26.57 | <0.001 |
| *Bull Breed* | 1223 | 6 | Belgian Blue | 39 (12%) | 112 (12%) | 0.86 | 0.43, 1.71 | 0.66 |
|  |  |  | Holst Friesian /Red HF | 180 (56%) | 539 (60%) | 1.00 |  |  |
|  |  |  | Fleckvieh | 46 (14%) | 123 (14%) | 4.31 | 1.02, 18.10 | 0.046 |
|  |  |  | Other pure breeds** | 53 (17%) | 120 (13%) | 1.50 | 0.57, 3.96 | 0.42 |
|  |  |  | Crossbreeds | 3 (1%) | 8 (1%) | 2.70 | 0.47, 15.62 | 0.27 |
| *Source of Bull* | 1115 | 14 | AI | 150 | 750 | 1.00 |  |  |
|  |  |  | Bull in herd | 51 (25%) | 164 (18%) | 1.33 | 0.63, 2.82 | 0.46 |

* Brown Swiss, Limousin, Limpurger, Pinzgau, Charolais, MRIJ, Montbéliarde, Abondance, Aubrac, Jersey, Eastern Flemish, Salers, Maine Anjou, Normande

**Brown Swiss, Limousin, Limpurger, Pinzgau, Charolais, MRIJ, Montbéliarde, Abondance, Scandinavian Roodbont, Aubrac, Angus, Blanc Bleu, Maine Anjou, Normande, Prog Federat Eur Pie, Blonde d’Aquitaine, Aure et St Girons Ca
