# Supplementary material for: Calf-Level Factors Associated with Bovine Neonatal Pancytopenia – A Multi-Country Case-Control Study
Source: PLoS One. 2013 Dec 2;8(12):e80619. doi: 10.1371/journal.pone.0080619 (PMC3846664; doi:10.1371/journal.pone.0080619)
Supplement: Table S5 — Results of descriptive and univariable analysis of dam vaccination variables. (DOCX) [file pone.0080619.s005.docx]

*Table S5 Results of descriptive and univariable analysis of dam vaccination variables (n=1296)*

| **Variable** | **n** | **% missing** | **Variable category** | **No. cases (%)** | **No. controls**  **(%)** | **Matched odds ratio (mOR)** | **95% confidence interval** | **Wald test p value** |
| --- | --- | --- | --- | --- | --- | --- | --- | --- |
| *BVD* *vaccination* | 1295 | 0.1 | No | 26 | 140 | 1.00 |  |  |
|  |  |  | Yes | 314 (92%) | 815 (85%) | 8.81 | 3.40, 22.82 | <0.001 |
| *No. mths before calving BVD vaccinated* | 1108 | 15 | 0-3 | 59 (19%) | 162 (20%) | 1.14 per change in level | 0.87, 1.50 | 0.35 |
|  |  |  | >3.5-6 | 51 (16%) | 158 (20%) |  |  |  |
|  |  |  | >6.5-9 | 35 (11%) | 90 (11%) |  |  |  |
|  |  |  | >9.5-12 | 37 (12%) | 100 (13%) |  |  |  |
|  |  |  | >13-75 | 131 (42%) | 285 (36%) |  |  |  |
| *BVD vacc within 12 mths of calving* | 1108 | 15 | < 12 mths | 182 | 510 | 1.00 |  |  |
|  |  |  | > 12 mths | 131 (42%) | 285 (36%) | 1.59 | 0.72, 3.49 | 0.25 |
| *No. BVD vaccination doses* | 1284 | 1 | 0 | 26 (8%) | 143 (15%) | 4.61 per change in level | 3.44, 6.17 | <0.001 |
|  |  |  | 1-3 | 63 (19%) | 346 (37%) |  |  |  |
|  |  |  | 4-6 | 188 (55%) | 395 (42%) |  |  |  |
|  |  |  | 7-11 | 62 (18%) | 61 (6%) |  |  |  |
| *Dam PregSure vaccinated* | 1296 | 0 | No | 36 | 241 | 1.00 |  |  |
|  |  |  | Yes | 304 (89%) | 715 (75%) | 16.23 | 6.95, 37.93 | <0.001 |
| *No. doses PregSure* | 1296 | 0 | 0 | 36 (11%) | 241 (25%) | 1.00 |  |  |
|  |  |  | 1-2 | 58 (17%) | 240 (25%) | 7.17 | 2.93, 17.53 | <0.001 |
|  |  |  | 3-4 | 190 (56%) | 346 (36%) | 31.02 | 12.54, 76.73 | <0.001 |
|  |  |  | 5-8 | 51 (15%) | 75 (8%) | 42.11 | 15.23, 116.42 | <0.001 |
|  |  |  | unknown doses | 5 (2%) | 54 (6%) | 1.35 | 0.30, 6.13 | 0.70 |
| *No. mths before calving PregSure vaccinated* | 935 | 28 | >1-3 | 3 (1%) | 9 (1%) | 1.43 per change in level | 0.89, 2.28 | 0.14 |
|  |  |  | >3-6 | 7 (2%) | 30 (5%) |  |  |  |
|  |  |  | >6-12 | 39 (13%) | 97 (15%) |  |  |  |
|  |  |  | >12-24 | 173 (58%) | 356 (56%) |  |  |  |
|  |  |  | >24-36 | 64 (21%) | 128 (20%) |  |  |  |
|  |  |  | >36-75 | 13 (4%) | 16 (3%) |  |  |  |
| *PregSure vacc within 12 mths of calving* | 935 | 28 | <=12 mth | 49 | 136 | 0.46 | 0.18, 1.16 |  |
|  |  |  | >12 mths | 250 (84%) | 500 (79%) | 1.00 |  | 0.099 |
| *Rispoval BVD* | 1284 | 1 | No | 325 | 913 | 1.00 |  |  |
|  |  |  | Yes | 14 (4%) | 32 (3%) | 1.96 | 0.17, 23.01 | 0.59 |
| *Rispoval RS-BVD* | 1287 | 1 | No | 329 | 936 | Non-convergence |  |  |
|  |  |  | Yes | 10 (3%) | 12 (1%) |  |  |  |
| *Rispoval 3 (RS, PI3, BVD)* | 1286 | 1 | No | 299 | 824 | 1.00 |  |  |
|  |  |  | Yes | 39 (12%) | 124 (13%) | 0.87 | 0.33, 2.31 | 0.78 |
| *Bovilis BVD* | 1287 | 1 | No | 235 | 644 | 1.00 |  |  |
|  |  |  | Yes | 104 (31%) | 304 (32%) | 2.14 | 0.73, 6.23 | 0.17 |
| *Bovidec BVD* | 1284 | 1 | No | 313 | 889 | 1.00 |  |  |
|  |  |  | Yes | 25 (7%) | 57 (6%) | 4.00 | 0.93, 17.20 | 0.063 |
| *Mucosiffa BVD* | 1290 | 0.5 | No | 317 | 907 | 1.00 |  |  |
|  |  |  | Yes | 22 (6%) | 44 (5%) | 2.91 | 0.67, 12.71 | 0.15 |
| *Mucobovin BVD* | 1287 | 1 | No | 337 | 942 | No within group variation |  |  |
|  |  |  | Yes | 2 (1%) | 6 (1%) |  |  |  |
| *Combinations of BVD Vaccines* | 1273 | 2 | no BVD vaccination | 26 (8%) | 140 (15%) | 0.10 | 0.03, 0.30 | <0.001 |
|  |  |  | PregSure only | 126 (37%) | 295 (32%) | 1.00 | - | - |
|  |  |  | PregSure + other BVD vaccine(s) | 177 (52%) | 408 (44%) | 3.18 | 1.26, 8.07 | 0.015 |
|  |  |  | Other BVD vaccine (single) | 6 (2%) | 75 (8%) | 0.13 | 0.03, 0.54 | 0.005 |
|  |  |  | Other BVD vaccines combined | 3 (1%) | 17 (2%) | 0.09 | 0.004, 1.95 | 0.13 |
| *Sequences of BVD Vaccines* | 1263 | 3 | no BVD vaccination | 26 (8%) | 140 (15%) | 0.10 | 0.03, 0.29 | <0.001 |
|  |  |  | PregSure only | 126 (37%) | 295 (32%) | 1.00 | - | - |
|  |  |  | PregSure > other BVD vaccine(s) | 122 (36%) | 293 (32%) | 1.92 | 0.64, 5.77 | 0.24 |
|  |  |  | Other BVD vaccine(s) > PregSure | 32 (10%) | 70 (8%) | 5.52 | 1.26, 24.14 | 0.023 |
|  |  |  | Other BVD > PregSure > other(s) | 21 (6%) | 38 (4%) | 5.69 | 1.21, 26.78 | 0.028 |
|  |  |  | PregSure > other BVD > PregSure | 1 (0.3%) | 0 | - | - |  |
|  |  |  | Other BVD vaccine (single) | 6 (2 %) | 75 (8%) | 0.11 | 0.03, 0.47 | 0.003 |
|  |  |  | Other BVD vaccines combined | 3 (0.9%) | 15 (2%) | 0.08 | 0.003, 2.12 | 0.13 |
| *Bluetongue vaccination* | 1256 | 3 | No | 34 | 139 | 1.00 |  |  |
|  |  |  | Yes | 299 (90%) | 784 (85%) | 2.80 | 1.25, 6.27 | 0.013 |
| *IBR* *vaccination* | 1237 | 5 | No | 215 | 599 | 1.00 |  |  |
|  |  |  | Yes | 111 (34%) | 312 (34%) | 2.76 | 0.78, 9.81 | 0.12 |
| *Rota/ coronavirus vaccination* | 1230 | 5 | No | 257 | 726 | 1.00 |  |  |
|  |  |  | Yes | 71 (22%) | 176 (20%) | 1.57 | 0.64, 3.87 | 0.33 |
| *Other vaccinations** | 1230 | 5 | No | 247 | 682 | 1.00 |  |  |
|  |  |  | Yes | 78 (24%) | 223 (25%) | 0.96 | 0.43, 2.12 | 0.92 |
| *No. diseases vaccinated against apart from BVD** | 1171 | 10 | 0 | 24 (8%) | 82 (10%) | 1.41 per change in level | 1.02, 1.94 | 0.037 |
|  |  |  | 1 | 108 (35%) | 326 (38%) |  |  |  |
|  |  |  | 2 | 117 (38%) | 260 (30%) |  |  |  |
|  |  |  | 3 | 19 (6%) | 68 (8%) |  |  |  |
|  |  |  | 4 | 32 (10%) | 101 (12%) |  |  |  |
|  |  |  | 5 | 11 (4%) | 23 (3%) |  |  |  |

*ringworm, clostridial diseases, botulism, parainfluenza 3 (PI3), pasteurella, bovine respiratory syncytial virus (BRSV), lungworm, *Escherichia coli*, Q fever, Chlamydophila abortus, mastitis (*E. coli, Staphylococcus aureus*)
