# Supplementary material for: Calf-Level Factors Associated with Bovine Neonatal Pancytopenia – A Multi-Country Case-Control Study
Source: PLoS One. 2013 Dec 2;8(12):e80619. doi: 10.1371/journal.pone.0080619 (PMC3846664; doi:10.1371/journal.pone.0080619)
Supplement: Table S6 — Number of PregSure doses by lactation number of cow. (DOCX) [file pone.0080619.s006.docx]

*Table S6 Number of PregSure doses by lactation number of cow (n=1296)*

|  | PregSure doses | | | | | |
| --- | --- | --- | --- | --- | --- | --- |
| Lactation | 0 | 1-2 | 3-4 | 5-8 | Unknown* | Total |
| First | 154 (49%) | 115 (37%) | 33 (10%) | 4 (1%) | 9 (3%) | 315 |
| Second | 43 (14%) | 102 (34%) | 133 (44%) | 9 (3%) | 12 (4%) | 299 |
| three plus | 80 (12%) | 81 (12%) | 370 (54%) | 113 (17%) | 38 (6%) | 682 |
| Total | 277 (21%) | 298 (23%) | 536 (41%) | 126 (10%) | 59 (5%) | 1,296 |

* reported to have received PregSure vaccination but number of doses was unknown
