# Supplementary material for: Calf-Level Factors Associated with Bovine Neonatal Pancytopenia – A Multi-Country Case-Control Study
Source: PLoS One. 2013 Dec 2;8(12):e80619. doi: 10.1371/journal.pone.0080619 (PMC3846664; doi:10.1371/journal.pone.0080619)
Supplement: Table S7 — Combinations of BVD vaccines. (DOCX) [file pone.0080619.s007.docx]

*Table S7. Combinations of BVD vaccines n=1273 (2% missing observations)*

| **Category** | **Case** | **Control** | **Total** |
| --- | --- | --- | --- |
| no BVD vaccination | 26 (8%) | 140 (15%) | 166 (13%) |
| PregSure only | 126 (37%) | 295 (32%) | 421 (33%) |
| Rispoval BVD only | 0 | 2 (0.2%) | 2 (0.2%) |
| Rispoval RS-BVD only | 0 | 1 (0.1%) | 1 (0.1%) |
| Rispoval 3 only | 0 | 12 (1%) | 12 (1%) |
| Bovilis BVD only | 4 (1%) | 45 (5%) | 49 (4%) |
| Bovidec BVD only | 0 | 9 (1%) | 9 (1%) |
| Mucosiffa/Vacoviron BVD only | 2 (0.6%) | 6 (0.6%) | 8 (1%) |
| PregSure + Rispoval BVD | 13 (4%) | 30 (3%) | 43 (3%) |
| PregSure + Rispoval RS-BVD | 8 (2%) | 11 (1%) | 19 (1%) |
| PregSure + Rispoval 3 | 22 (7%) | 59 (6%) | 81 (6%) |
| PregSure + Bovilis BVD | 77 (23%) | 198 (21%) | 275 (22%) |
| PregSure + Bovidec BVD | 16 (5%) | 27 (3%) | 43 (3%) |
| PregSure + Mucosiffa/Vacoviron | 16 (5%) | 27 (3%) | 43 (3%) |
| PregSure + Mucobovin | 1 (0.3%) | 3 (0.3%) | 4 (0.3%) |
| PregSure + Rispoval 3 + Bovilis BVD | 12 (4%) | 32 (3%) | 44 (3%) |
| Rispoval 3 + Bovilis BVD | 2 (0.6%) | 11 (1%) | 13 (1%) |
| Bovilis BVD + Bovidec BVD | 0 | 4 (0.4%) | 4 (0.3%) |
| Bovilis BVD + Vacoviron | 1 (0.3%) | 1 (0.1%) | 2 (0.2%) |
| Rispoval 3 + Bovidec BVD | 0 | 1 (0.1%) | 1 (0.1%) |
| PregSure + Rispoval BVD + Bovilis BVD + Bovidec BVD | 1 (0.3%) | 0 | 1 (0.1%) |
| Pregsure + Rispoval 3 + Bovilis BVD + Bovidec BVD | 1 (0.3%) | 1 (0.1%) | 2 (0.2%) |
| Pregsure + Bovilis BVD + Bovidec BVD | 5 (1%) | 9 (1%) | 14 (1%) |
| PregSure + Rispoval 3 + Bovidec BVD | 2 (0.6%) | 6 (0.6%) | 8 (0.6%) |
| PregSure + Rispoval RS-BVD + Mucosiffa | 2 (0.6%) | 0 | 2 (0.2%) |
| PregSure + Mucosiffa + Rispoval 3 | 0 | 2 (0.2%) | 2 (0.2%) |
| PregSure + Vacoviron + Mucobovin | 1 (0.3%) | 3 (0.3%) | 4 (0.3%) |
|  | 338 | 935 | 1273 |
