# Supplementary material for: Calf-Level Factors Associated with Bovine Neonatal Pancytopenia – A Multi-Country Case-Control Study
Source: PLoS One. 2013 Dec 2;8(12):e80619. doi: 10.1371/journal.pone.0080619 (PMC3846664; doi:10.1371/journal.pone.0080619)
Supplement: Table S8 — Sequences of BVD vaccines. (DOCX) [file pone.0080619.s008.docx]

*Table S8. Sequences of BVD vaccines (n= 1263, 3% missing observations)*

| **Category** | **Case** | **Control** | **Total** |
| --- | --- | --- | --- |
| no BVD vaccination | 26 (8%) | 140 (15%) | 166 (13%) |
| PregSure only | 126 (37%) | 295 (32%) | 412 (33%) |
| Rispoval BVD only | 0 | 2 (0.2%) | 2 (0.2%) |
| Rispoval RS-BVD only | 0 | 1 (0.1%) | 1 (0.1%) |
| Rispoval 3 only | 0 | 12 (1%) | 12 (1%) |
| Bovilis BVD only | 4 (1%) | 45 (5%) | 49 (4%) |
| Bovidec BVD only | 0 | 9 (1%) | 9 (0.7%) |
| Mucosiffa BVD only | 2 (0.6%) | 6 (0.7%) | 8 (0.6%) |
| PregSure > Rispoval BVD | 12 (4%) | 27 (3%) | 39 (3%) |
| Rispoval BVD > PregSure | 1 (0.3%) | 3 (0.3%) | 4 (0.3%) |
| PregSure > Rispoval RS-BVD | 2 (0.6%) | 7 (0.8%) | 9 (0.7%) |
| Rispoval RS-BVD > PregSure | 4 (1%) | 4 (0.4%) | 8 (0.6%) |
| Rispoval RS-BVD > PregSure > Rispoval RS-BVD | 2 (0.6%) | 0 | 2 (0.2%) |
| Rispoval 3 > PregSure | 22 (7%) | 59 (6%) | 81 (6%) |
| PregSure > Bovilis BVD | 75 (22%) | 194 (21%) | 269 (21%) |
| Bovilis BVD > PregSure | 2 (0.6%) | 4 (0.4%) | 6 (0.5%) |
| PregSure > Bovidec BVD | 15 (4%) | 27 (3%) | 42 (3%) |
| Bovidec BVD > PregSure | 1 (0.3%) | 0 | 1 (0.1%) |
| PregSure > Mucosiffa | 13 (4%) | 27 (3%) | 40 (3%) |
| Mucosiffa/Vacoviron > PregSure | 2 (0.6%) | 0 | 1 (0.1%) |
| PregSure > Vacoviron > PregSure | 1 (0.3%) | 0 | 1 (0.1%) |
| PregSure > Mucobovin | 1 (0.3%) | 3 (0.3%) | 4 (0.3%) |
| Rispoval 3 > PregSure > Bovilis BVD | 12 (4%) | 32 (3%) | 44 (3%) |
| Rispoval 3 > Bovilis BVD | 2 (0.6%) | 11 (1%) | 13 (1%) |
| Bovidec BVD > Bovilis BVD | 0 | 2 (0.2%) | 2 (0.2%) |
| Bovilis BVD > Vacoviron | 1 (0.3%) | 1 (0.1%) | 2 (0.2%) |
| Rispoval 3 > Bovidec BVD | 0 | 1 (0.1%) | 1 (0.1%) |
| Rispoval BVD > PregSure > Bovidec BVD > Bovilis BVD | 1 (0.3%) | 0 | 1 (0.1%) |
| Rispoval 3 > PregSure > Bovilis BVD > Bovidec BVD > Bovilis BVD | 1 (0.3%) | 1 (0.1%) | 2 (0.2%) |
| PregSure > Bovidec BVD > Bovilis BVD | 4 (1%) | 5 (0.5%) | 9 (0.7%) |
| Bovilis BVD > PregSure > Bovidec BVD | 1 (0.3%) | 2 (0.2%) | 3 (0.2%) |
| Rispoval 3 > PregSure > Bovidec BVD | 1 (0.3%) | 3 (0.3%) | 4 (0.3%) |
| Rispoval RS-BVD > PregSure > Mucosiffa | 2 (0.6%) | 0 | 2 (0.2%) |
| PregSure > Mucobovin > Vacoviron | 0 | 3 (0.3%) | 3 (0.2%) |
| Mucobovin > Vacoviron > PregSure > Mucobovin > Vacoviron | 1 (0.3%) | 0 | 1 (0.1%) |
| Total | 337 | 926 | 1263 |
