# Supplementary material for: Calf-Level Factors Associated with Bovine Neonatal Pancytopenia – A Multi-Country Case-Control Study
Source: PLoS One. 2013 Dec 2;8(12):e80619. doi: 10.1371/journal.pone.0080619 (PMC3846664; doi:10.1371/journal.pone.0080619)
Supplement: Table S9 — Multivariable model Stata output. (DOCX) [file pone.0080619.s009.docx]

*Table S9 Multivariable model Stata output (n=1296)*

Stata command: clogit ccstatus Pregsureever##i.DamParity3cat Pregsureever##ColosNotDam i.milk3, group(Farm) or vce(robust) base

| Exposure variable |  | Adjusted matched odds ratio (amOR) | 95% confidence interval | Wald test P value |
| --- | --- | --- | --- | --- |
| Dam PregSure vaccination | Unvaccinated | 1.0 |  |  |
|  | Vaccinated | 17.8 | 2.4, 134.4 | 0.005 |
| Lactation no. | 1 | 1.0 |  |  |
|  | 2 | 0.5 | 0.1, 1.6 | 0.23 |
|  | 3+ | 0.7 | 0.2, 2.4 | 0.60 |
| Colostrum from different cow(s) | No | 1.0 |  |  |
|  | Yes | 30.5 | 2.1, 440.5 | 0.012 |
| Raw milk from dam only | No | 1.0 |  |  |
|  | Yes | 3.4 | 1.6, 7.5 | 0.002 |
| Interaction between dam PregSure vaccination and lactation no. | Vaccinated & lactation 2  Vaccinated & lactation 3+ | 4.8  7.4 | 1.1, 20.7  1.9, 28.9 | 0.034  0.004 |
| Interaction between dam PregSure vaccination and colostrum from different cow(s) | Vaccinated & colostrum from different cow(s) Yes | 0.1 | 0.01, 0.95 | 0.046 |
