# Supplementary material for: Calf-Level Factors Associated with Bovine Neonatal Pancytopenia – A Multi-Country Case-Control Study
Source: PLoS One. 2013 Dec 2;8(12):e80619. doi: 10.1371/journal.pone.0080619 (PMC3846664; doi:10.1371/journal.pone.0080619)
Supplement: Questionnaire S1 — (DOCX) [file pone.0080619.s010.docx]

Text S1. Questionnaire

Key to variable categories

ID descriptive variable

Calving observed excluded variable due to low number of responses, differences in interpretation between countries, or to anonymise the data

Suckled dam potential risk factor variable

**Bovine neonatal pancytopenia questionnaire**

Questions refer to the previous 12 months except where specifically stated

1. Farm details

| Country |  |
| --- | --- |
| Owner |  |
| Farm Number , ID |  |
| Address |  |
| Telephone |  |
| Email (owner): |  |
| Veterinarian |  |
| Address |  |
| Telephone |  |
| Email (vet): |  |
| Production type |  |

**Signalement of BNP calf and control calves:**

| ID | BNP CASE calf | Control calf-1 | Control calf-2 | Control calf-3 | Control calf-4 |
| --- | --- | --- | --- | --- | --- |
| Breed |  |  |  |  |  |
| Sex |  |  |  |  |  |
| Birthdate |  |  |  |  |  |
| Calving observed | Yes/no | Yes/no | Yes/no | Yes/no | Yes/no |
| Calved isolated or in group? | Individ / group | Individ / group | Individ / group | Individ / group | Individ / group |
| Twin? BNP? | Yes/no  Yes/no | Yes/no  Yes/no | Yes/no  Yes/no | Yes/no  Yes/no | Yes/no  Yes/no |

**(in grey questions which the veterinarian might know)**

| Date of clinical onset or first treatment date |  |
| --- | --- |
| Condition at clinical onset | Alert/depressed/coma |
| Age (between 0 and 28 days) | Age in days: |
| Multiple bleeding on the skin and / or melena and / or petechiae in the mucous membranes | Yes/no (if yes: please tick)  □ multiple bleeding skin □ melena □ petechia in mucous membranes |
| Hematology shows pancytopenia | Yes/no |
| Leukocytes (<2.0 x 10^9^ / L)  (if possible state the measured number) | Number: |
| Thrombocytopenia = (<100 x 10^9^ / L)  (if possible state the measured number) | Number: |
| BVD PCR result  Pooled samples?  Individual samples?  If positive, which BVDV type? | Positive /negative / not performed  Yes/no  Yes/no |
| BTV PCR result | Positive /negative / not performed |
| Condition if dead calf:  Age at death:  Bleeding history  Bone marrow histology | _______days  Yes/no  BNP yes/ BNP no/ not performed |

**In case of death of the case-calf**

| Date of death |  |
| --- | --- |
| Euthanasia: yes/no | Spontaneous: yes/no |
| Necropsy: yes/no  If yes, please include results |  |

**Control calves**: any clinical signs at time of sampling:

|  | Control calf-1 | Control calf-2 | Control calf-3 | Control calf-4 |
| --- | --- | --- | --- | --- |
| Clinical signs of bleeding | Yes/no | Yes/no | Yes/no | Yes/no |
| diarrhoea | Yes/no | Yes/no | Yes/no | Yes/no |
| Lung problems | Yes/no | Yes/no | Yes/no | Yes/no |
| Any other clinical signs?  If yes, please state: | Yes/no | Yes/no | Yes/no | Yes/no |
| Any treatments? If yes, please state: | Yes/no | Yes/no | Yes/no | Yes/no |

**Colostrum (this case and its controls) circle as appropriate**

| Criteria | BNP CASE calf | Control calf-1 | Control calf-2 | Control calf-3 | Control calf-4 |
| --- | --- | --- | --- | --- | --- |
| ID |  |  |  |  |  |
| Born at what time of day  Morning 6am-12pm  Afternoon 12pm to 6pm  Evening 6pm to 10pm  Night 10pm to 6am | Morning  Afternoon  Evening  Night | Morning  Afternoon  Evening  Night | Morning  Afternoon  Evening  Night | Morning  Afternoon  Evening  Night | Morning  Afternoon  Evening  Night |
| Suckled Dam within first 12 hours | - yes  - Possible, not sure  - no | - yes  - Possible, not sure  - no | - yes  - Possible, not sure  - no | - yes  - Possible, not sure  - no | - yes  - Possible, not sure  - no |
| Separation from dam within 24 h => how long between birth and first colostrum administration: | ____hrs | ____hrs | ____hrs | ____hrs | ____hrs |
| - how many times did calf receive colostrum within 24 hours?  - Quantity of colostrum received in total  - colostrum obtained from cow/s different from dam?  - own or different farm?  - from multiple cows (pooled)?  - frozen colostrum | _________  _________  Yes/no  Own/differ.  Yes/no  Yes/no | _________  _________  Yes/no  Own/differ.  Yes/no  Yes/no | _________  _________  Yes/no  Own/differ.  Yes/no  Yes/no | _________  _________  Yes/no  Own/differ.  Yes/no  Yes/no | _________  _________  Yes/no  Own/differ.  Yes/no  Yes/no |
| **If frozen colostrum is used:** (if info available)  - from own or different farm?  - ID of cow  - lactation number  - same vaccination as herd?  - BVD vaccination of cows if different from herd:  - other vaccination of cows if different from herd: | Own/differ.  Yes/no | Own/differ.  Yes/no | Own/differ.  Yes/no | Own/differ.  Yes/no | Own/differ.  Yes/no |
|  |  |  |  |  |  |
| Criteria | BNP CASE calf | Control calf-1 | Control calf-2 | Control calf-3 | Control calf-4 |
| **If pooled colostrum is used:**  - colostrum of birth dam included?  - BVD vaccination of cows if different from herd:  - other vaccination of cows if different from herd: | yes/no/not known | yes/no/not known | yes/no/not known | yes/no/not known | yes/no/not known |
| Criteria | BNP CASE calf | Control calf-1 | Control calf-2 | Control calf-3 | Control calf-4 |
| **If colostrum replacer is used:** (artificial colostrum)  - colostrum replacer only?  - additional to dam colostrum?  - additional to frozen colostrum  - if colostrum replacer: product name:  - quantity of colostrum replacer | Yes/no  Yes/no  Yes/no | Yes/no  Yes/no  Yes/no | Yes/no  Yes/no  Yes/no | Yes/no  Yes/no  Yes/no | Yes/no  Yes/no  Yes/no |

**Milk feeding (tick as appropriate)**

|  | Case-calf | Control calf-1 | Control calf-2 | Control calf-3 | Control calf-4 |
| --- | --- | --- | --- | --- | --- |
| Milk powder |  |  |  |  |  |
| Raw milk |  |  |  |  |  |
| Raw milk from dam |  |  |  |  |  |
| Bulk milk |  |  |  |  |  |
| Milk from cows with high SCC or clinical  mastitis |  |  |  |  |  |
| Withdrawn/discarded milk (e.g. from cows  treated for mastitis) |  |  |  |  |  |
| Other |  |  |  |  |  |

1. Dam of current BNP case and control calves

(A maximum of 2 cases and 8 controls per farm; if two cases, please fill in this section twice)

|  | Dam of BNP CASEcalf | Dam of Control calf-1 | Dam of Control calf-2 | Dam of Control calf-3 | Dam of Control calf-4 |
| --- | --- | --- | --- | --- | --- |
| ID |  |  |  |  |  |
| Date of birth (day, month, yr) |  |  |  |  |  |
| Breed |  |  |  |  |  |
| Lactation number |  |  |  |  |  |
| 305 day milk yield during previous lactation |  |  |  |  |  |
| Estimated breeding value for milk |  |  |  |  |  |
| Calf resulted from embryo-transfer into this cow? If yes, give genetic dam ID if available? | Yes/no | Yes/no | Yes/no | Yes/no | Yes/no |
| Was the dam born on the farm? | Yes/no | Yes/no | Yes/no | Yes/no | Yes/no |
| Purchased animal?  If yes: date of purchase:  Farm of origin:  Already pregnant at purchase? | Yes/no  Yes/no | Yes/no  Yes/no | Yes/no  Yes/no | Yes/no  Yes/no | Yes/no  Yes/no |
| Rearing at another farm? If yes, in which period was she at the other farm? | Yes/no | Yes/no | Yes/no | Yes/no | Yes/no |
| Gave birth to a previously affect. calf with similar clinical picture? | Yes/no | Yes/no | Yes/no | Yes/no | Yes/no |

**Outcome of the previous calves within the last years :**

|  |  | Dam of BNP CASE calf | Dam of Control calf-1 | Dam of Control calf-2 | Dam of Control calf-3 | Dam of Control calf-4 |
| --- | --- | --- | --- | --- | --- | --- |
| Calf  n-1: | Bleeding disorder⁭  Clinical signs  Death  Age at death | Yes/no;  Yes/no; < 4 wks/ later  Yes/no  ___________d | Yes/no;  Yes/no; < 4 wks/ later  Yes/no  ___________d | Yes/no;  Yes/no; < 4 wks/ later  Yes/no  ___________d | Yes/no;  Yes/no; < 4 wks/ later  Yes/no  ___________d | Yes/no;  Yes/no; < 4 wks/ later  Yes/no  ___________d |
| Calf  n-2: | Bleeding disorder⁭  Clinical signs  Death  Age at death | Yes/no;  Yes/no; < 4 wks/ later  Yes/no  ___________d | Yes/no;  Yes/no; < 4 wks/ later  Yes/no  ___________d | Yes/no;  Yes/no; < 4 wks/ later  Yes/no  ___________d | Yes/no;  Yes/no; < 4 wks/ later  Yes/no  ___________d | Yes/no;  Yes/no; < 4 wks/ later  Yes/no  ___________d |

**Vaccinations of the dam of case-calf**

|  | **Vaccine product name(s)** | **dates**  *from 2005 onwards*  *(day, month, year)* |
| --- | --- | --- |
| **BTV** |  |  |
|  |  |  |
|  |  |  |
| **BVD** | BVD vaccinated: yes/no  How many months before calving?  How often has the dam been vaccinated against BVD (including the first double booster vaccination) (lifetime number of vaccinations)?  (please enter vaccine product names/years used in rows below) |  |
|  |  |  |
|  |  |  |
|  |  |  |
| **Diarrhoea (Rota/ Corona)** |  |  |
|  |  |  |
| **IBR** |  |  |
|  |  |  |
|  |  |  |
| **Others** |  |  |
|  |  |  |
|  |  |  |

**Any treatments** (during gestation of case or in early lactation)**/ treatment at drying off**

|  |
| --- |

**Vaccination of dams of control calves:**

|  | **Dam of control calf:** | | | | **Differences and additional vaccinations (please state the control calf number)** |
| --- | --- | --- | --- | --- | --- |
|  | **Calf1** | **Calf2** | **Calf3** | **Calf4** |  |
| **BTV** | Yes/no | Yes/no | Yes/no | Yes/no |  |
| **BVD**  **Months before calving?**  **How often?** | Yes/no  _____  _____ | Yes/no  _____  _____ | Yes/no  _____  _____ | Yes/no  _____  _____ |  |
| **Diarrhoea (Rota/ Corona)** | Yes/no | Yes/no | Yes/no | Yes/no |  |
| **IBR** | Yes/no | Yes/no | Yes/no | Yes/no |  |
| **Others** | Yes/no | Yes/no | Yes/no | Yes/no |  |

**Any treatments** (during gestation of control calves or in early lactation)**/ treatment at drying off; please state the control calf number**

|  |
| --- |

1. Bull of current case and controls

|  | BNP CASE calf | Control calf-1 | Control calf-2 | Control calf-3 | Control calf-4 |
| --- | --- | --- | --- | --- | --- |
| ID/ AI number |  |  |  |  |  |
| Name of bull |  |  |  |  |  |
| Breed |  |  |  |  |  |
| Bull in herd |  |  |  |  |  |
| AI |  |  |  |  |  |
| Was the bull sire of previously affected calf/calves? |  |  |  |  |  |
